# Climate Change and Diabetes Mellitus - Emerging Global Public Health Crisis: Observational Analysis

**DOI:** 10.12669/pjms.40.4.8844

**Published:** 2024

**Authors:** Sultan Ayoub Meo, Anusha Sultan Meo

**Affiliations:** 1 Prof. Sultan Ayoub Meo, Department of Physiology, College of Medicine, King Saud University, Riyadh, Saudi Arabia; 2 Dr. Anusha Sultan Meo, The School of Medicine, Medical Sciences and Nutrition, University of Aberdeen, Scotland, United Kingdom

**Keywords:** Climate change, Weather conditions, Temperature, Type 2 Diabetes

## Abstract

Climate change is the most pressing challenge of the 21st century. It’s immediate impacts on the environment are extreme weather conditions such as heatwaves, storms, rains, floods, sealevel rise, the disruption of crops, agricultural systems, water, vector-borne diseases, and ecosystems. The weather-related disasters disturbed the natural biological environment and dislocated millions of people from their homes. The extreme weather conditions caused the deaths of about two million people and $4.3 trillion in economic loss over the past half a century, and 90% of deaths were reported from developing countries. It has also been predicted that between 2030 and 2050, climate change is presumed to cause about 250,000 additional deaths per annum. The rapid rise in temperatures, frequencies of heat waves, wildfires, storms, and other weather extremes conditions could affect human health in many ways. The one-degree Celsius rise in outdoor temperature causes over 100,000 new cases of diabetes mellitus per annum. Climate change compromised body metabolism, vasodilation, sweating, insulin resistance and cause Type-2 diabetes mellitus and gestational diabetes Mellitus.

## INTRODUCTION

The environment and climate conditions have a profound influence on human health. The presence of human civilization alters this landscape. The swift urbanization, industrialization and population growth are the contributors towards environmental pollution and climate change and now humans’ own existence is at risk due to the repercussions of their actions on the earth. The survival of humans depends on the accessibility of clean air, water, food, and normal thermal environments. If these planetary boundaries are exceeded, then the survival of the ecosystem is threatened. Climate change is caused by human activities, and it has disturbed the entire environment, ecology, and natural cycles such as the water cycle, food chain etc. This has further impacts on the lives of humans, animals, and plants. The effects also include the new types of diseases and the impact on existing chronic debilitating diseases like diabetes mellitus.^1^,^2^

The rapid change in the climate affects the environment in many ways, for instance, warming of the environment may force the people, and animal species to migrate to higher latitudes where temperatures are more conducive for their sustainable survival. Similarly, as the level of the sea rises, saltwater causes intervention in the freshwater system and some species relocate or are eradicated. Climate change markedly affects ecosystems, species, and their development, and worsens the living environment.^1^,^3^

Climate change, driven by the increase in environmental pollution, and greenhouse gas emissions, is altering the Earth’s climate system to unprecedented levels. The consequences of global warming are multifaceted, with rising temperatures, changing precipitation patterns, and increased frequency of extreme weather events are the most conspicuous outcomes. Moreover, climate change is greatly impacting human health in various ways, including acute and chronic illness, disabilities and deaths from extreme weather events such as heatwaves, storms, rains, floods, the disruption of food systems, water and vector-borne diseases and neurological health issues These conditions not only affect the natural ecosystem of the earth but develop various acute and chronic diseases with long-lasting illnesses, complications, disabilities and deaths.^4^

### Climate Change and Global Morbidity and Mortality

According to the United Nations, World Meteorological Organization (WMO), 11,778 weather-related disasters have occurred from 1970 to 2021. The severe weather conditions caused about two million deaths, and $4.3 trillion in economic loss over the period of the last half of the century. It was further reported that 90% of fatalities were due to disasters that took place in developing nations. In the year 2021, it was found that worldwide more than 50,000 deaths per annum were due to these weather-allied conditions.^5^ The World Health Organization estimated that between 2030 and 2050, climate change is expected to cause about 250,000 additional deaths per annum due to malnutrition, malaria, diarrhoea, and heat stress. The damage costs to health are estimated to be about USD 2-4 billion per year by 2030.^4^

### Climate Change and Diabetes Mellitus

Worldwide, the number of people with diabetes mellitus is about 537 million, aged 20-79 years. This figure is estimated to increase to 643 million by 2030 and 783 million by 2045, over three in four adults with diabetes live in low- and middle-income countries. Moreover, diabetes caused about 6.7 million deaths in the year 2021. It shows that one person dies every five seconds due to complications of diabetes mellitus. Furthermore, diabetes mellitus causes an economic loss of about 966 billion dollars in health expenditure.^6^ The prevalence of diabetes mellitus could be affected by urbanization, industrialization, environmental pollution, and urban development contributing to climate change.^7^

**Fig.1:**
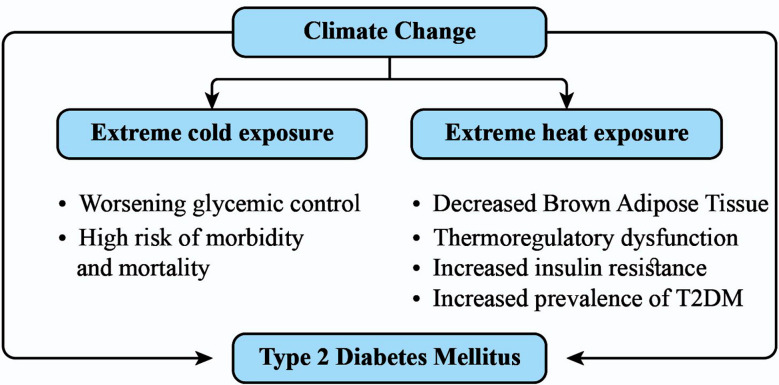
Climate change and Type 2 Diabetes Mellitus

### Summer Seasons, High Temperatures and Diabetes Mellitus

The complex relationship between climate change and metabolic health has recently been clarified by influencing correlations between ambient temperatures and T2DM. Prolonged heat waves and the human body’s exposure to elevated temperatures can lead to heat strokes. This causes the body to struggle with maintaining its core temperature within the normal range (around 98.6°F or 37°C). This results in heat-related illnesses like heat exhaustion. The body’s regulatory mechanisms, such as sweating and vasodilation become overwhelmed, leading to potential health risks. It affects blood sugar levels.^8^ The high ambient temperatures are linked with an increased risk for T2DM. Heat stress can cause several physiological changes that have a significant impact on the processes in glucose metabolism, insulin sensitivity, and beta-cell activity. The rapid development of T2DM occurs due to long-term heat exposure which negatively affects the body’s capacity to control blood sugar levels.^9^ Blauw et al., 2017^10^ investigated the relationship between outdoor temperature and the incidence of diabetes mellitus. The authors adjusted the confounding factors and found that an increase in the incidence of Type-1 or Type-2 diabetes was linked with an elevated temperature. The authors further reported that a 1°C rise in outdoor temperature was linked with over 100,000 new cases of diabetes per year.^10^

Similarly, Valdés et al., 2019^11^ found that the ambient temperature has an association with the prevalence of dysglycaemia and insulin resistance. The upsurge in insulin resistance and blood glucose levels was linked with an increase in the ambient temperatures and moderately.^12^

**Table-I:** Effects of climate change and temperatures on the incidence of diabetes mellitus.

Author name and year	Country and type of study	Weather Condition	Study outcomes
Blauw et al., 2017^10^	Retrospective USA	Hot temperature	A rise in 1°C temperature enhanced the incidence of diabetes by 0.314 (CI: 0.194-0.434). The glucose intolerance was intensified by 0.170% (CI: 0.107-0.234%) per one °C rise in temperature.
Vasileiou et al. 2018^17^	Retrospective study, China	Hot temperature	GDM risk was increased at hot temperatures in the first and second trimesters of pregnancy.
Molina-Vega et al. 2020^18^	Retrospective study	Hot temperatures	High temperatures for 14-28 days increased the risk of GDM in high-temperature months of March-August, but not during months in which temperature was low Sept-Feb.
Teyton et al., 2023^19^	Retrospective study, USA	Both temperature	The risk of GDM increased with extremely low temperature during gestational weeks 20-24 & and extremely high temperature for 11-16 weeks.
Natur Sarya 2022^20^	-	High temperature	High temperatures affect insulin sensitivity and cause an increased incidence of diabetes mellitus.

The pathophysiological events that facilitate the relationship between increased ambient temperatures and insulin resistance may be triggered by dehydration and its following impact on insulin resistance and hepatic gluconeogenesis.^13^ Additionally, the changes in the temperature have an impact on the brown adipose tissues.^14^ All these facts support the hypothesis that climate change can cause insulin resistance and diabetes mellitus.

Qian et al., 2023^15^ conducted a retrospective study and found that extreme temperatures during the various trimesters of pregnancy could cause gestational diabetes mellitus (GDM). The authors reported that during the first and second trimesters of pregnancy, the risk of GDM was augmented in the summer with elevated temperatures; however, in the second trimester, the risk of GDM increased in winter with low temperatures.

The literature demonstrated that GDM risk was significantly elevated during the months of summer than in the months of winter, and GDM risk was increased with elevated temperatures.^16^,^17^ Molina-Vega et al.^18^ recruited 2374 women, among them GDM was diagnosed in about 473 (20.0%) of the females. It was also observed that seasonal differences in the occurrence of GDM were 24.40% in summer and 15.60% in autumn. Similarly, Teyton et al.^19^ reported that GDM risk was increased with extremely hot temperatures at weeks 11-16 and extremely low temperatures during gestational weeks 20-24. All these fats support the hypothesis that extreme changes in temperatures have a relationship with increased incidence of T2DM and GDM.

### High Temperatures, Dehydration and T2DM

Elevated temperatures can cause water loss through sweating and that can lead to dehydration. The dehydration can negatively impact body functions. Normal body fluids and adequate hydration are highly essential for the maintenance and regulation of various physiological and biochemical functions.^21^ Roussel et al.,^22^ performed a study on 3615 participants and identified 565 (15.62%) cases of hyperglycemia and concluded that water intake was independently and inversely related to the risk of developing hyperglycemia. Similarly, Trainor et al.,^23^ identified that severe dehydration was linked with the onset of diabetes mellitus. The possible mechanisms for this high blood glucose were the hormones related to the hypothalamic-pituitary axis and the renin-angiotensin-aldosterone system (RAAS).^24^

### Pathophysiology of High Temperature and Diabetes Mellitus

There are multiple mechanisms and responses involved between exposure to heat and elevated temperature and T2DM. Extensive and prolonged exposure to hot temperatures causes vasodilation and sweating leading to fluid loss, dehydration, electrolyte imbalances and hemoconcentration. Dehydration and increased blood flow to the skin impair insulin signalling and glucose disposal, by inhibiting cellular insulin action and reducing blood flow to insulin-sensitive tissues, respectively.^25^ Dehydration promotes insulin resistance by interfering with signalling downstream of phosphoinositide 3-kinase, including hyperosmotic inhibition of insulin-induced protein kinase B activation.^26^ Dehydration increases vasopressin levels, which can stimulate gluconeogenesis in the liver and promote insulin resistance by acting on the liver, adipose tissue, pancreas, and the pituitary gland. Heat and elevated temperatures affect the main metabolic pathways involved in the development of insulin resistance and Type-2 diabetes.^13^

## CONCLUSIONS

The extreme change in climate conditions increasing the frequencies of temperatures, heat waves, storms, wildfires, heavy precipitation, and other weather conditions can affect human health. The one-degree Celsius rise in outdoor temperature causes over 100,000 new cases of diabetes mellitus per annum. Climate change compromised body metabolism, vasodilation, sweating, insulin resistance and diabetes mellitus. The regional, national and global societies and political communities rethink the policies to minimize environmental pollution and combat climate change and diabetes mellitus.

### Authors’ Contributions:

**SAM:** Writing and editing the manuscript.

**ASM:** Literature review, checking, and analysis.

All authors have read & approved the manuscript.

## References

[1] Climate Change Impacts The United States Environmental Protection Agency (EPA).

[2] Ratter-Rieck JM, Roden M, Herder C (2023). Diabetes, and climate change:current evidence and implications for people with diabetes, clinicians, and policy stakeholders. Diabetologia.

[3] Settele JR, Scholes R, Betts S, Bunn P, Leadley D, Nepstad JT, Field CB, Barros VR (2014). Terrestrial and Inland Water Systems. In:Climate Change 2014:Impacts, Adaptation and Vulnerability. Part A:Global and Sectoral Aspects. Contribution of Working Group II to the Fourth Assessment Report of the Intergovernmental Panel on Climate Change.

[4] World Health Organization (WHO) Climate change and health.

[5] Aljazeera Climate change causes 2m deaths in 50 years;poor suffer most:UN.

[6] IDF Diabetes Atlas, Diabetes around the world in 2021.

[7] Chen K, Wolf K, Breitner S, Gasparrini A, Stafoggia M, Samoli E (2018). Two-way effect modifications of air pollution and air temperature on total natural and cardiovascular mortality in eight European urban areas. Environ Int.

[8] CDC. Centres for Disease Control and Prevention. 2020 Managing Diabetes in the Heat.

[9] Penn LDI (2023). People with Type 2 Diabetes and Extreme Temperatures.

[10] Blauw LL, Aziz NA, Tannemaat MR, Blauw CA, de Craen AJ, Pijl H (2017). Diabetes incidence and glucose intolerance prevalence increase with higher outdoor temperatures. BMJ Open Diabetes Res Care.

[11] Valdés S, Doulatram-Gamgaram V, Lago A, Torres FG, Badía-Guillén R, Olveira G (2019). Ambient temperature and prevalence of diabetes and insulin resistance in the Spanish population:Diabet.es study. Eur J Endocrinol.

[12] Tucker P, Gilliland J (2007). The effect of season and weather on physical activity:a systematic review. Public Health.

[13] Vanhaecke T, Perrier ET, Melander O (2020). A journey through the early evidence linking hydration to metabolic health. Ann Nutr Metab.

[14] Lee P, Smith S, Linderman J, Courville AB, Brychta RJ, Dieckmann W (2014). Temperature-acclimated brown adipose tissue modulates insulin sensitivity in humans. Diabetes.

[15] Qian N, Xu R, Wei Y, Li Z, Wang Z, Guo C (2023). Influence of temperature on the risk of gestational diabetes mellitus and hypertension in different pregnancy trimesters. Sci Total Environ.

[16] Booth GL, Luo J, Park AL, Feig DS, Moineddin R, Ray JG (2017). Influence of environmental temperature on the risk of gestational diabetes. Canadian Med Assoc J.

[17] Vasileiou V, Kyratzoglou E, Paschou SA, Kyprianou M, Anastasiou E (2018). The impact of environmental temperature on the diagnosis of gestational diabetes mellitus. Eur J Endocrinol.

[18] Molina-Vega M, Gutiérrez-Repiso C, Muñoz-Garach A, Lima-Rubio F, Morcillo S, Tinahones FJ (2020). Relationship between environmental temperature and the diagnosis and treatment of gestational diabetes mellitus:An observational retrospective study. Sci Total Environ.

[19] Teyton A, Sun Y, Molitor J, Chen JC, Sacks D, Avila C (2023). Examining the Relationship Between Extreme Temperature, Microclimate Indicators, and Gestational Diabetes Mellitus in Pregnant Women Living in Southern California. Environ Epidemiol.

[20] Sarya N, Damri O, Agam G (2022). The Effect of Global Warming on Complex Disorders (Mental Disorders, Primary Hypertension, and Type 2 Diabetes). Int J Environ Res Public Health.

[21] Popkin BM, D'Anci KE, Rosenberg IH (2010). Water, hydration, and health. Nutr Rev.

[22] Roussel R, Fezeu L, Bouby N, Balkau B, Lantieri O, Alhenc-Gelas F (2011). Low water intake and risk for new-onset hyperglycemia. Diabetes Care.

[23] Trainor JL, Glaser NS, Tzimenatos L, Stoner MJ, Brown KM, McManemy JK (2023). Clinical and Laboratory Predictors of Dehydration Severity in Children with Diabetic Ketoacidosis. Ann Emerg Med.

[24] Johnson EC, Bardis CN, Jansen LT, Adams JD, Kirkland TW, Kavouras SA (2017). Reduced water intake deteriorates glucose regulation in patients with type 2 diabetes. Nutr Res.

[25] Schliess F, Reissmann R, Reinehr R, vom Dahl S, Häussinger D (2004). Involvement of integrins and SRC in insulin signalling toward autophagic proteolysis in rat liver. J Biol Chem.

[26] Schliess F, Häussinger D (2003). Cell volume and insulin signaling. Int Rev Cytol.

